# Supplementary material for: Causal Relationship between Aging and Anorexia Nervosa: A White-Matter-Microstructure-Mediated Mendelian Randomization Analysis
Source: Biomedicines. 2024 Aug 16;12(8):1874. doi: 10.3390/biomedicines12081874 (PMC11351342; doi:10.3390/biomedicines12081874)
Supplement: Supplementary file 1 [file biomedicines-12-01874-s001.zip › Supplementary Figure/Figure S9/Leaveoneout plot of MR analysis for ubm-b-1947 to AN.pdf]

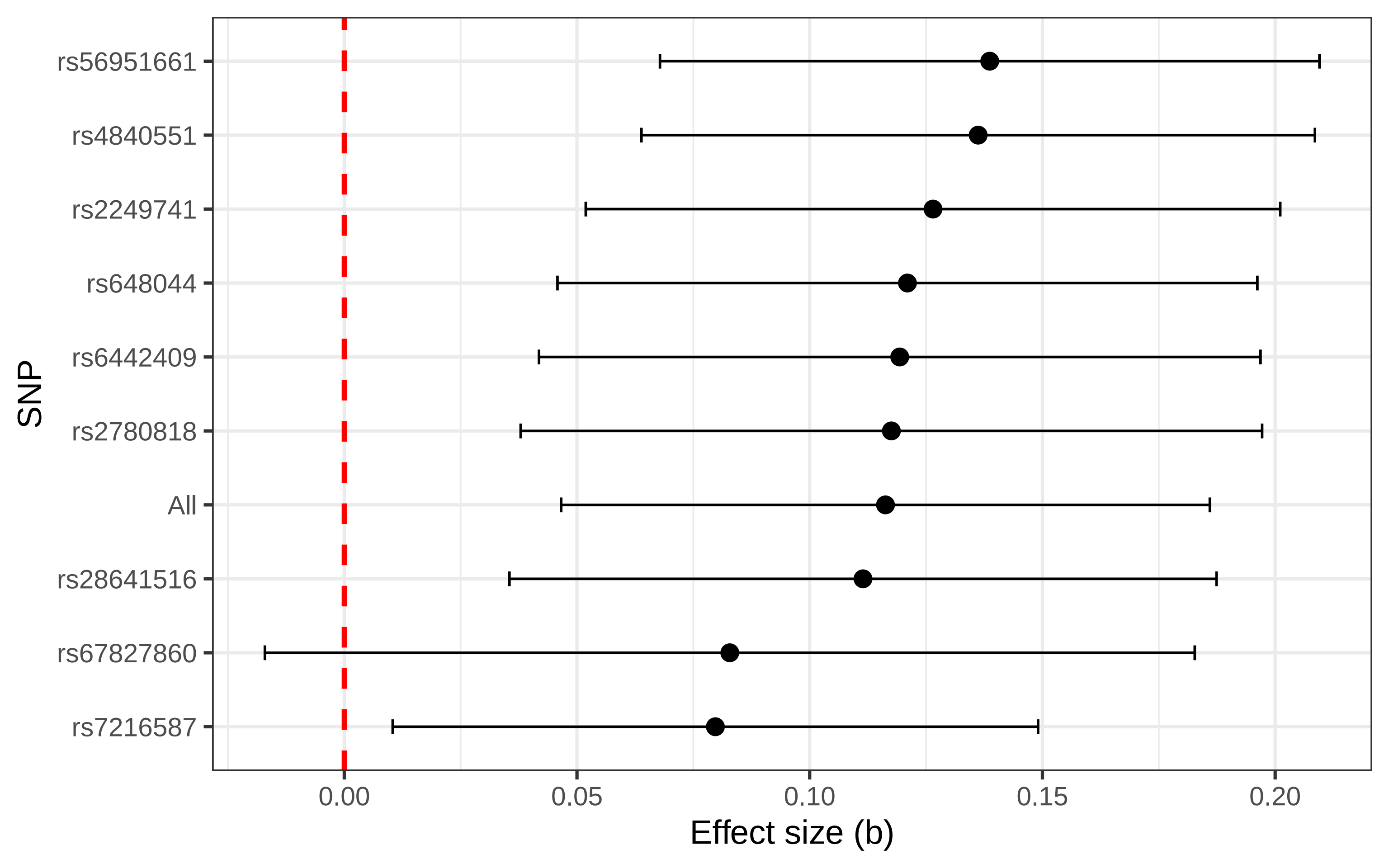

SNP
rs56951661
rs4840551
rs2249741
rs648044
rs6442409
rs2780818
All
rs28641516
rs67827860
rs7216587
0.00
0.05
0.10
0.15
0.20
Effect size (b)
